# A pilot study for treatment of severe COVID-19 pneumonia by aerosolized formulation of convalescent human immune plasma exosomes (ChipEXO™)

**DOI:** 10.3389/fimmu.2022.963309

**Published:** 2022-11-09

**Authors:** Fethi Gül, Zeynep Burcin Gonen, Olcay Y. Jones, Neslihan Pakize Taşlı, Gökmen Zararsız, Ekrem Ünal, Aykut Özdarendeli, Fikrettin Şahin, Ahmet Eken, Semih Yılmaz, Musa Karakukçu, Oğuz Kaan Kırbaş, Nur Seda Gökdemir, Batuhan Turhan Bozkurt, Yusuf Özkul, Burçin Doruk Oktay, Muhammet Ali Uygut, Ismail Cinel, Mustafa Çetin

**Affiliations:** ^1^ Department of Anesthesiology and Reanimation, Division of Critical Care Medicine, School of Medicine, Marmara University, Istanbul, Türkiye; ^2^ Betül-Ziya Eren Genome and Stem Cell Center (GENKOK), Kayseri, Türkiye; ^3^ Division of Rheumatology, Department of Medicine, George Washington University School of Medicine and Health Sciences, Washington, DC, United States; ^4^ Department of Genetics and Bioengineering, Faculty of Engineering and Architecture, Yeditepe University, İstanbul, Türkiye; ^5^ Department of Biostatistics, Faculty of Medicine, Erciyes University, Kayseri, Türkiye; ^6^ Department of Pediatrics, Division of Pediatric Hematology, Faculty of Medicine, Erciyes University, Kayseri, Türkiye; ^7^ Faculty of Medicine, Vaccine Research and Development Application and Research Center, Erciyes University, Kayseri, Türkiye; ^8^ Department of Biology, Faculty of Science, Erciyes University, Kayseri, Türkiye; ^9^ Institute of Health Sciences, Department of Medical Biochemistry, Erciyes University, Kayseri, Türkiye; ^10^ Faculty of Medicine, Erciyes University, Kayseri, Türkiye; ^11^ Department of Anesthesiology and Reanimation, Division of Critical Care Medicine, School of Medicine, Marmara University, İstanbul, Türkiye; ^12^ Vaccine Research and Development Application and Research Center, Erciyes University, Kayseri, Türkiye

**Keywords:** COVID-19, Coronavirus disease, Severe acute respiratory syndrome-coronavirus-2, SARS-CoV-2, exosomes, convalescent plasma

## Abstract

This is a single-center prospective, open-label, single arm interventional study to test the safety and efficacy of recently described ChipEXO™ for severe COVID-19 pneumonia. The ChipEXO™ is a natural product derived from convalescent human immune plasma of patients recovered from moderate COVID-19 infection. In September 2021, 13 patients with pending respiratory failure were treated with ChipEXO™ adapted for aerosolized formulation delivered *via* jet nebulizer. Patients received 1-5x10^10^ nano vesicle/5 mL in distilled water twice daily for five days as an add-on to ongoing conventional COVID-19 treatment. The primary endpoint was patient safety and survival over a 28-day follow-up. The secondary endpoint was longitudinal assessment of clinical parameters following ChipEXO™ to evaluate treatment response and gain insights into the pharmacodynamics. ChipEXO™ was tolerated well without any allergic reaction or acute toxicity. The survival rate was 84.6% and 11 out of 13 recovered without any sequel to lungs or other organs. ChipEXO™ treatment was effective immediately as shown in arterial blood gas analyses before and two hours after exosome inhalation. During the 5 days of treatment, there was a sustainable and gradual improvement on oxygenation parameters: i.e. respiratory rate (RR) [20.8% (P < 0.05)], oxygen saturation (SpO_2_) [6,7% (P < 0.05)] and partial pressure of oxygen to the fraction of inspired oxygen (PaO_2_/FiO_2_) [127.9% (P < 0.05)] that correlated with steep decrease in the disease activity scores and inflammatory markers, i.e. the sequential organ failure assessment (SOFA) score (75%, p < 0.05), C-reactive protein (46% p < 0.05), ferritin (58% p = 0.53), D-dimer (28% p=0.46). In conclusion, aerosolized ChipEXO™ showed promising safety and efficacy for life-threatening COVID-19 pneumonia. Further studies on larger patient populations are required to confirm our findings and understand the pathophysiology of improvement toward a new therapeutic agent for the treatment of severe COVID-19 pneumonia.

## Introduction

The coronavirus disease 2019 (COVID-19) pandemic has posed an unprecedented need for new antiviral therapeutics that are safe, effective, and readily available for the need to treat large populations. Severe acute respiratory syndrome coronavirus 2 (SARS-CoV-2), the causative agent of COVID-19, is an airborne disease targeting the lung epithelial cells resulting in viral pneumonia in about 20% of the infected (1). This is the major cause of mortality —so far, 6 million worldwide— due to the development of acute respiratory distress syndrome (ARDS) which involves inflammatory cascades and endothelial damage (2, 3). It is characterized by disruption of lung epithelial-endothelial barrier integrity, resulting in thickening of alveolar walls, inflammation, and fibrosis, which leads to impaired alveolar-capillary gas exchange and impaired immune response (4, 5). Conventional treatments to limit viral load or decrease inflammation have been limited as mortality rates remain over 50% among patients with severe COVID-19 pneumonia (6, 7).

Since the early days of the pandemic, many countries have been engaging in large-scale operations to collect and store convalescent serum from the survivors (8). This is considered a historical remedy, dating back to the 19^th^ century, to provide passive immunity when needed. In fact, successful applications of convalescent plasma treatment (CPT) have been reported during the epidemics by the members of Coronoviridea, SARS, and MERS in the last two decades (9, 10). Similar observations have been published recently for applications of CPT for severe COVID-19 infection (11–13). Traditionally, convalescent plasma has been used to deliver passive immunity through the antiviral antibody content to reduce the viral load. Recently, the immunotherapeutic and biologic activities of convalescent plasma, have been focused on harnessing plasma content for extracellular vesicles (EV) including exosomes for the treatment of COVID-19 by intravenous infusion (14–16). EVs are small message-bearing vesicles ubiquitously produced by all known types of cells. Exosomes are a subtype of EVs of endosomal origin, and range between 30 to 200 nm in size. Through their protein and RNA cargo, exosomes can convey biological information to other cells upon uptake *via* endocytosis (17). Recently, we reported the antiviral efficacy of convalescent human immune plasma-derived exosome (ChipEXO™) against SARS-CoV-2 using preclinical models (18). Based on the omics data, the cargo content includes a range of proteins, lipids, RNA and DNA, etc, but it is free of antibodies.

In this pilot study, we have studied ChipEXO™ as an inhaled agent for treatment of patients with COVID-19 admitted to the intensive care unit (ICU) for pending respiratory failure. The results observed are promising and warrant further research to explore underlying pathophysiology of improvement.

## Patients and methods

This is a prospective, open-label, case controlled clinical study conducted at Marmara University Anesthesiology and Reanimation ICU in collaboration with the laboratories of Erciyes University upon proper approvals by the Clinical Studies Ethics Committees and Turkish Ministry of Health. ICU patients with polymerase chain reaction (PCR) positive COVID-19 associated ARDS were enrolled after signed informed consent during ICU stay for respiratory support in the month of September 2021. The Delta variant was the most common dominant strain during the study period. The enrollment criteria included respiratory rate >30 breaths/min, SpO_2_ <92% on room air at sea level, and a ratio of arterial partial pressure of oxygen to fraction of inspired oxygen (PaO_2_/FiO_2_) <300 mm Hg. Patients who have serious general conditions, such as severe organ dysfunction and initially required mechanical ventilation at admission were excluded. All patients were on ongoing standard COVID-19 care per published guidelines by the Turkish Ministry of Health (19) that included dexamethasone 6 mg by mouth once daily (or equivalent methyl prednisolone), favipiravir 1600 mg by mouth twice daily for a day (loading dose) then 600 mg twice daily (maintanence), and enoxaparin 0.4 ml subcutaneously once daily. The primary endpoint was the safety of ChipEXO™ treatment and survival during the 28 day follow-up. The secondary endpoints included improvement of respiratory parameters, clinical symptoms and laboratory parameters.

### Clinical follow up parameters

Patients were closely monitored for vital signs, laboratories as well as Sequential Organ Failure Assessment (SOFA) scores (20), Acute Physiology and Chronic Health Evaluation (APACHE) II, heart rate, acidosis, consciousness, oxygenation, and respiratory rate (HACOR) scores (21, 22). Thoracic computed tomography (CT) findings were classified according to COVID-19 Reporting and Data System (CO-RADS) (21).

### ChipEXO™ collection, isolation and application

Convalescent plasma was obtained from the survivors of COVID-19 according to national regulations set forth by the Turkish Ministry of Health (19). Exosomes were isolated from convalescent plasma and prepared for usage as described previously (18) at Erciyes University’s good manufacturing practices (GMP) laboratories. Physical characterization including size distribution and concentration of exosomes was measured *via* nanoparticle tracking analysis (NTA) using Nano-sight NS300 (Malvern Instruments, Malvern, UK). Samples were diluted in phosphate-buffered solution (PBS) to contain 25–200 particles in a frame and examined by 15 captures of 20 second each. Threshold levels were selected for each sample according to the manufacturer’s instructions (23). The diameter analysis of exosomes was performed by scanning electron microscopy (Zeiss GEMINI 500). ChipEXO^TM^ was screened for contamination (BacTAlert system) before application. Residual dextran levels was monitored by using Fourier Transform Infrared Spectroscopy (FTIR).

The exosome stock solution was diluted with distilled water to have 20-60 particles visible in the NTA camera. The final dilute (1-5 x 10^10^ Nano-vesicle in 5 mL) ChipEXO™ was delivered by jet nebulizer as an inhaled agent twice daily for five days. None of the patients experienced any side effects that were attributable to administration of ChipEXO™ inhalation within this period.

### Statistical analysis

Histogram, q-q plots were examined and Shapiro-Wilk’s test was used to assess the data normality. Wilcoxon t and Friedman tests were used for within group comparisons. Nemenyi test was applied for post-hoc comparisons. Analyses were conducted on data from 13 subjects using R 4.0.1 (24) and TURCOSA (Turcosa Analytics Ltd. Co., Türkiye, www.turcosa.com.tr) software. A *P* value less than 5% was considered as statistically significant.

## Results

Thirteen patients who were admitted to ICU for respiratory support were studied. As shown in **Table 1A**, Demographics included mean age of 55.9 years (range: 39-74), 8 males (61.5%). The comorbid conditions included diabetes mellitus (38.5%), hypertension (30.7%), coronary heart disease (15.4%), asthma (7.7%), chronic obstructive pulmonary disease (7.7%), and history of cancer (7.7%). The most common symptoms at admission were cough (84.6%), shortness of breath (69.2%), and fever (61.5%). There was median 7 [Interquartile range (IQR, 5-8)]; days between detection of PCR positivity and admission to ICU. At the time of admission, median APACHE II score was median 12 (IQR, 9-17) and at admission. HACOR score was 6 (IQR, 5-8). Thoracic computed tomography findings were consistent with COVID-19 CO-RADS group 5 in all patients.

**Table 1A:** Demographics and clinical status before chipEXO^TM^ treatment.

Total number of subjects enrolled	13
Age* ± SD	55.9±11.2
Male (%)	8 (61.5)
**Previous coexisting disease, subject number (%)**	
Hypertension	4 (30.7)
Diabetes mellitus	5 (38.5)
Cancer history	1 (7.7)
Asthma & COPD	1 (7.7)
CHD	2 (15.4)
**Symptoms, subject number (%)**	
Fever	8 (61.5)
Cough	11 (84.6)
Shortness of breath	9 (69.2)
Sputum	3 (23.1)
Diarrhea	1 (7.7)
Sore throat	3 (23.1)
**Disease severity at enrollment, median (range** **)**	
Days since positive PCR	7 (5-8)
APACHE-II score	12 (9-17)
SOFA score	8 (6.5-9.0)
P SILI HACOR score	6 (5-8)
CO-RADS score	5 (5-5)

As summarized in **Table 1B**, The clinical course of patients during 5 days of ChipEXO™ treatment is as following: All patients required high flow nasal oxygen (HFNO) therapy at admission and four (30.7%) progressed to require non-invasive mechanical ventilation (NIMV) and five patients (38.5%) required mechanical ventilation (MV). The median duration of HFNO therapy, NIMV and MV was 4 days (IQR, 1-8), 4 days (IQR, 2-8) and 9 days (IQR, 6.5-10.5), respectively. Two (15.4%) out of 13 enrolled expired from bacterial infections and sepsis after a median duration of ICU stay for 13.5 days (IQR, 12-13.5) 12 and 13.5 days of ICU stay. The leading cause of death was secondary bacterial infections and sepsis in both patients. Remaining 11 (84.6%) survivors discharged successfully after a median duration of ICU and hospital stay of 10 days (IQR, 9-12) and 18 days (IQR, 12-19), respectively. Mortality at 28 days was remarkably lower (15.4%) with a shorter duration of ICU stay and a greater probability of discharge alive (84.6%) among those treated with ChipEXO™ in comparison to national average.

**Table 1B:** Clinical follow up & final outcomes after chipEXO^TM^ treatment.

**Clinical Follow Up**	
Number subjects on HFNO > NIMV > MV (%)	13(100)> 4(30.7)> 5(38.5)
Median days of HFNO > NIMV > MV (range )	4(1-8)> 5(1-11.5)> 9(6.5-10.5)
**Final Outcome at 28 days follow-up**	
Number deceased (%)	2(15.4)
Total days ICU stay of deceased, median (IQR range )	13.5 (12-13.5)
Number survivors (%)	11(84.6)
Total days of ICU stay of survivors, median (IQR range )	10 (9-12)
Total days of hospital stay of survivors, median (IQR range )	18 (12-19)

Age, mean in years; SD, standard deviation; PCR, polymerase chain reaction; CHD, coronary heart disease; SOFA, Sequential Organ Failure Assessment scores; APACHE, Acute Physiology and Chronic Health Evaluation II scores; HACOR, Heart Rate, Acidosis, Consciousness, Oxygenation, Respiratory Rate scores; CO-RADS, Thoracic computed tomography classification for COVID-19; HFNO, High Flow nasal oxygen therapy; NIMV, Non-invasive mechanical ventilation; MV, mechanical ventilation.

**Figure 1:**
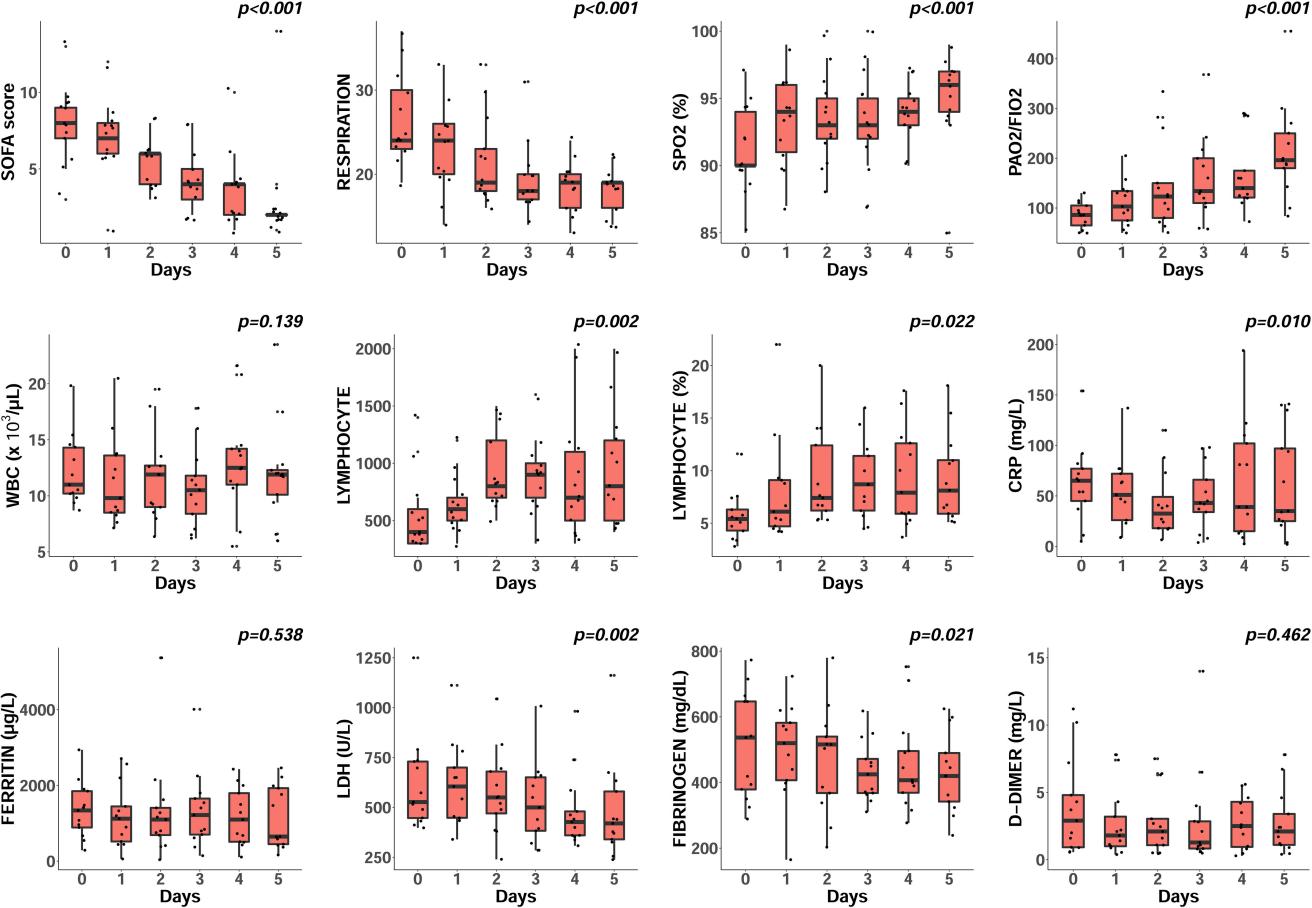
Impact of ChipEXO^TM^ on clinical and laboratory parameters. Subjects received twice daily aerosolized ChipEXO^TM^ treatment for 5 days. Results are shown as scattered boxplots to demonstrate the distribution of change for each parameter over time. P values, by Friedman test, indicate a within-subject comparison on six time points. There was a significant improvement in the respiratory rate, SOFA score and lymphocyte count. The SpO_2_ level and PaO_2_/FiO_2_ ratio dramatically increased, and CRP, LDH and fibrinogen significantly decreased during treatment. Abbreviations (normal range): WBC, white blood cell count; CRP, C-reactive protein; LDH, lactate dehydrogenase.

As shown in **Figure 1**, there was significant improvement in the respiratory rate [median 24.0 (IQR, 23.0-31.0) *vs* 19.0 (IQR, 15.0-19.5), p<0.001];, SOFA score [median 8.0 (IQR, 6.5-9.0) vs 2.0 (IQR, 2.0-2.0), p<0.001]; number of lymphocytes [median 400.0 (IQR, 300.0-650.0)/µl *vs* median 800.0 (IQR, 600.0-1250.0), p=0.002];, SpO_2_ level [median 90.0 (IQR, 89.5-94.0) *vs* 96.0 (IQR, 93.5-97.0), p=0.007];, and PaO_2_/FiO_2_ ratio [median 86.0 (IQR, 60.0-107.5) *vs* 196.0 (IQR, 161.5-260.0), p<0.001]; during 5 days of inhaled exosome, ChipEXO™, therapy. The levels of C-reactive protein (CRP), lactate dehydrogenase (LDH) and fibrinogen also significantly decreased during this period.

**Figure 2:**
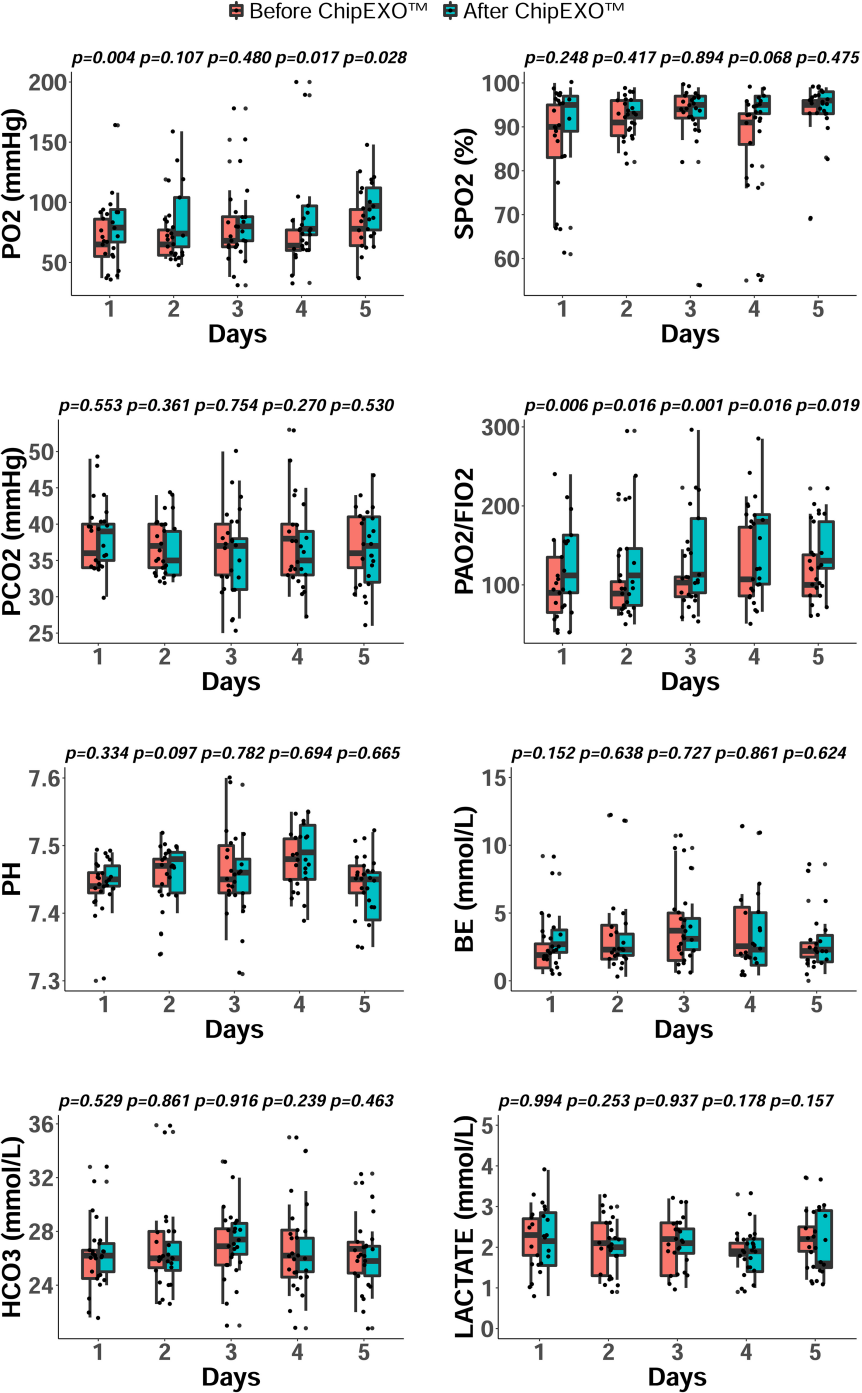
Arterial Blood Gas parameters during ChipEXO^TM^ Treatment. The respiratory parameters of oxygenation and ventilation were followed daily before (red) and two hours post (green) ChipEXO^TM^ treatment for 5 days. Results are shown as grouped and scattered boxplots for the distribution and change in each of the parameter studied. P values, by Wilcoxon test, display the levels of significance between pre- and post- treatment at a given time point. Abbreviations (normal range): pO_2_, partial pressure of oxygen (83-108 mmHg); pCO_2_, partial pressure of carbon dioxide (35-45 mmHg); sPO_2_, oxygen saturation (95%-99%); BE, base excess (-3 to 3 mmol/L); HCO3, bicarbonate (22-28 mmol/L); pH (7.35-7.45), lactate (0.5-1.5 mmol/L).

As shown in **Figure 2**, when arterial blood gas analyses were compared before exosome inhalation and two hours after exosome inhalation, there was a rapid and robust improvement in gas exchange and oxygenation parameters for PO_2_ and PaO_2_/FiO_2_. A statistically significant increase was observed for PO_2_ levels on days 4 and 5, and for PaO_2_/FiO_2_ ratio on all days before and two hours after exosome inhalation (P < 0.05). For PO_2_, the area under the curve (AUC) before and after ChipEXO™ treatment was 352.50 (314.50-406.25) and 416.00 (357.25-515.50), [P <0, 05] on day 1 and day 5, respectively. Similarly, for PaO_2_/FiO_2_ levels, AUC before and after ChipEXO™ treatment was 505.00 (387.25-683.25) on day 1 and 625.50 (578.00-804.00) on day 1 and day 5, respectively [P <0, 05]. Furthermore, the improvement of PO_2_ levels was sustainable and additive on each consequent day over a 5 day treatment; i.e. PO_2_ levels at 65.0 mmHg (49.0-89.0) and 78.0 mmHg (63.0-94.5), [P> 0.05] on day 1 and day 5, respectively. Improvement in PaO_2_/FiO_2_ was highly striking between the first and fifth day of inhaled exosome therapy at 90.0 (62.5-144.0) and 100 (80–164), respectively [P> 0.05]. The SPO_2_ levels also significantly improved between the first and fifth day of the treatment, i.e. 90.0% (80.0-95.5) on day 1 and 95.0% (92.5-96.5) on day 5, [P> 0.05], although, the immediate effect of ChipEXO™ on the levels of SPO_2_ within 2 hours of treatment was not statistically significant.

## Discussion

As of April 2022, the number of laboratory-confirmed patients with SARS-CoV-2 infection reached to half a billion world-wide causing more than six million deaths (https://covid19.who.int, last accessed on April 10, 2022 (2). So far, dexamethasone has been the only treatment that is readily available during the pandemic to provide significant impact. Although it is widely used as the standard of care in critically ill patients, the mortality from ARDS still remains high emphasizing the urgent need for development of affordable new therapeutic options (25).

We now report a novel therapeutic aerosol treatment composed of exosomes derived from immune plasma, ChipEXO™, against COVID19 pneumonia. Based on the omics studies, we have shown that the cargo content of ChipEXO™ differs from healthy control-derived preparation for miRNA expression and protein composition (18). In particular, ChipEXO™ is enriched for proteins associated with three main groups: immune system, microvasculature and somatic cells. For immune activation and modulation, terms such as “response to symbiont” (a.k.a. response to the virus), “cytolysis by a host of symbiont cells,” and “killing by a host of symbiont cells” included C4b-binding protein (C4BP) alpha and beta chains, apolipoprotein L1, histidine-rich glycoprotein, and prothrombin. The proteins under “molecular function” annotated five proteins under “complement binding” and four under “immunoglobulin binding,” with enrichment of 80.39-fold and 58.72-fold, respectively, in comparison to the expected number of proteins per the PANTHER reference list of the Homo sapiens gene database.

All 13 patients who were enrolled had impaired gas exchange and disturbed oxygenation and hypoxemia that are well-known for unfavorable clinical outcomes and poor survival rates (26–29). Furthermore, all 13 had abnormal biomarkers with elevated CRP, D-Dimer, LDH, fibrinogen, ferritin levels, and significantly higher SOFA, APACHE II, and HACOR risk scores and met the criteria for severe ARDS pneumonia due to their down-trending PaO_2_/FiO_2_ ratio and intensively infiltrated lung images.

Respiratory aerosol ChipEXO™ significantly improved the respiratory rate, PO_2_, SPO_2_, and PaO_2_/FiO_2_ from the first day of administration to the fifth day. After aerosol exosome administration, a hyper-acute respiratory improvement was consistently observed in arterial blood oxygenation and gas exchange parameters. All 11 patients survived, allowed a slow wean of oxygen support, a known predictive factor for recovery and eventual hospital discharge. This was in conjunction with significant improvement in patients’ clinical scores (SOFA, APACHE II, HACOR) and laboratory values (absolute lymphocyte number, d-dimer, CRP, fibrinogen, LDH and ferritin) during the 5 day treatment. The improvement of each parameter was sustainable and in parallel allowing a coherent course among the survivors, including those (n=5) who required a brief period of mechanical support. Reduction in inflammation markers during the course of ChipEXO™, as an add-on treatment to the conventional systemic treatment, suggests the importance of targeted treatment to break the cascading events leading to fetal outcomes from COVID-19 pneumonia. Based on the omics data, as well as, the results of d-dimer levels, the cargo content is likely to have biological activities on microvasculature.

In summary, this study showed a significant benefit of inhaled exosome therapy in the respiratory functions of critically ill COVID-19 patients. The treatment was safe and tolerated well; i.e. similar to our observation on preclinical murine model, there was no serious adverse effects, allergic or toxic reaction. Major limitations of this study include small numbers of patient enrollment and lack of a sham control. Nonetheless, considering the mortality rate of 58% that we experienced among the 20,000 hospitalized COVID-19 patients during the same time period at our institution, the survival rate accomplished in this pilot study is encouraging. This was not due to unbiased patient selection as evidenced by 5 progressing to require mechanical ventilation. Although the concept of exosome based treatment is not novel, targeted delivery of convalescent serum derived exosomes during the pandemic has not been reported. There is an ongoing research on the mechanism of improvement; based on our preclinical and omics data (18), the bioactivities of ChipEXO^TM^ are likely to be multifactorial and may include direct inhibition of viral propagation, immune modulation and promoting vascular health. Thus a cocktail of key cargo components may be necessary to initiate changes simultaneously for synergy and healing. If the results of future clinical trials on ChipEXO^TM^ is promising, further pharmacologic research is warranted on molecular pathways and feasibility of customized recombinant products. 

## Data availability statement

The original contributions presented in the study are included in the article/**Supplementary Material**. Further inquiries can be directed to the corresponding author.

## Ethics statement

The studies involving human participants were reviewed and approved by Clinical Studies Ethics Committees and Turkish Ministry of Health. The patients/participants provided their written informed consent to participate in this study.

## Author contributions

The experiment design of this study, the construction and analysis of the experiments were done by FG, NT, ZG, OYJ, and MÇ. Plasma samples collected by FG. Exosome production were performed by NT, ZG, NG, OK, BB, YÖ, MK, GZ, EU, and FŞ. ChipEXO^TM^ were administered by FG, MU, İC, BO, and SY. All authors were writing their own part of the manuscript. Grammar correction and final writing were done by OYJ and AE. All authors read and approved the final manuscript. All authors contributed to the article and approved the submitted version.

## Conflict of interest

The authors declare that the research was conducted in the absence of any commercial or financial relationships that could be construed as a potential conflict of interest.

## Publisher’s note

All claims expressed in this article are solely those of the authors and do not necessarily represent those of their affiliated organizations, or those of the publisher, the editors and the reviewers. Any product that may be evaluated in this article, or claim that may be made by its manufacturer, is not guaranteed or endorsed by the publisher.
