# Supplementary material for: A pilot study for treatment of severe COVID-19 pneumonia by aerosolized formulation of convalescent human immune plasma exosomes (ChipEXO™)
Source: Front Immunol. 2022 Nov 9;13:963309. doi: 10.3389/fimmu.2022.963309 (PMC9682905; doi:10.3389/fimmu.2022.963309)
Supplement: Supplementary file 1 [file Table_1.docx]

| **SUPPLEMENTARY TABLE 1. IMPACT OF CHIPEXO ON CLINICAL AND RESPIRATORY PARAMETERS DURING A 5 DAY TREATMENT COURSE** | | | | | | | | |
| --- | --- | --- | --- | --- | --- | --- | --- | --- |
| **Variables*** | **Initial^†^** | **Days After Administration‡** | | | | | ***p*** |  |
|  | **Day 0** | **Day 1** | **Day 2** | **Day 3** | **Day 4** | **Day 5** |  |  |
| **SOFA** | 8.0(6.5-9.0)^a^ | 7.0(6.0-8.0)^a^ | 6.0(4.0-6.0)^ab^ | 4.0(2.5-5.5)^b^ | 4.0(2.0-4.0)^b^ | 2.0(2.0-2.0)^b^ | **<0.001** |  |
| **FEVER** | 36.4(36.4-36.6) | 36.4(36.3-36.6) | 36.5(36.4-36.5) | 36.3(36.3-36.6) | 36.4(36.4-36.5) | 36.4(36.3-36.5) | 0.606 |  |
| **RESPIRATİON** | 24.0(23.0-31.0)^a^ | 24.0(19.5-26.0)^a^ | 19.0(18.0-25.0)^b^ | 18.0(17.0-20.5)^b^ | 19.0(16.0-20.0)^b^ | 19.0(15.0-19.5)^b^ | **<0.001** |  |
| **SPO2** | 90.0(86.5-94.0)^a^ | 94.0(90.5-96.0)^ab^ | 93.0(91.0-95.5)^ab^ | 93.0(91.5-96.0)^ab^ | 94.0(93.0-96.0)^ab^ | 96.0(93.5-97.0)^b^ | **0.001** |  |
| **PAO2/FİO2** | 86.0(60.0-107.5)^a^ | 103.0(70.0-135.5)^ab^ | 123.0(76.0-205.5)^bc^ | 134.0(106.0-208.5)^bc^ | 140.0(100.0-200.0)^bc^ | 196.0(161.5-260.0)^c^ | **<0.001** |  |
| **WBC** | 10500(9600-14450) | 9800(8400-13700) | 11900(8750-13100) | 10500(7700-12500) | 12500(10850-14350) | 11900(9750-12550) | 0.139 |  |
| [**LYMPHOCYTE**](https://tureng.com/tr/turkce-ingilizce/lymphocyte) **(#)** | 400.0(300.0-650.0)^a^ | 600.0(450.0-800.0)^ab^ | 800.0(700.0-1300.0)^b^ | 900.0(650.0-1050.0)^b^ | 700.0(450.0-1150.0)^ab^ | 800.0(600.0-1250.0)^b^ | **0.002** |  |
| [**LYMPHOCYTE**](https://tureng.com/tr/turkce-ingilizce/lymphocyte) **(**%**)** | 5.4(3.9-6.9)^a^ | 6.1(4.6-9.2)^ab^ | 7.4(5.8-13.2)^b^ | 8.7(6.0-11.7)^b^ | 7.9(6.9-12.9)^ab^ | 8.1(5.8-11.7)^b^ | **0.022** |  |
| **CRP** | 65.0(41.0-79.5) | 51.0(24.5-74.5) | 41.0(18.0-80.5) | 43.0(22.6-77.0) | 39.0(14.0-106.0) | 35.0(23.9-116.0) | **0.010** |  |
| **PCT** | 0.10(0.05-0.20) | 0.07(0.05-0.15) | 0.12(0.04-0.18) | 0.10(0.06-0.14) | 0.08(0.06-0.15) | 0.10(0.05-0.16) | 0.487 |  |
| **FERRİTİN** | 1340(781.0-1875) | 1123(483.0-1823.5) | 1100.0(551.5-1515.5) | 1219.0(659.5-1727.5) | 1100.0(506.5-1898.5) | 650.0(435.5-1959.0) | 0.538 |  |
| **LDH** | 527.0(439.5-733.0)^ab^ | 605.0(446.5-741.0)^a^ | 550.0(427.5-687.5)^ab^ | 500.0(351.5-655.5)^ab^ | 427.0(356.5-533.0)^ab^ | 420.0(333.0-607.0)^b^ | **0.002** |  |
| **FİBRİNOGEN** | 537.0(364.5-655.5)^a^ | 520.0(402.5-601.0)^a^ | 516.0(353.0-556.0)^a^ | 425.0(366.0-505.5)^b^ | 407.0(353.5-523.5)^b^ | 420.0(333.0-540.0)^b^ | **0.021** |  |
| **D-DİMER** | 2.90(0.91-6.00) | 1.80(0.94-3.75) | 2.10(0.80-4.67) | 1.28(0.82-3.43) | 2.60(0.90-5.05) | 2.10(1.00-4.00) | 0.462 |  |
| *** Values are expressed median (1^st^-3^rd^ quartiles). Statistically significant *P* values are shown in bold. † Just Before First Administration, ‡ Just Before Daily Sequential administration** | | | | | | | | |

| **SUPPLEMENTARY TABLE 2. Arterial Blood Gas Parameters Before And 2 hours post CHİPEXO™ during a 5 day- treatment course** | | | | | | | |
| --- | --- | --- | --- | --- | --- | --- | --- |
| **Variables** | **Day 1 (*n* = 13)** | **Day 2 (*n* = 13)** | **Day 3 (*n* = 13)** | **Day 4 (*n* = 13)** | **Day 5 (*n* = 13)** | **AUC** | ***p*** |
| **PO2** |  | | | | | | |
| **Before ChipEXO™** | 65.0(49.0-89.0) | 65.0(56.0-81.5) | 68.0(61.0-99.0) | 64.0(58.0-77.5) | 78.0(63.0-94.5) | 352.50(314.50-406.25) | 0.277 |
| **After ChipEXO™** | 79.0(61.5-96.0) | 74.0(60.5-111.0) | 80.0(65.5-90.0) | 78.0(70.5-101.0) | 97.0(74.5-114.0) | 416.00(357.25-515.50) | 0.291 |
| ***P*** | **0.004** | 0.107 | 0.480 | **0.017** | **0.028** | **0.009** |  |
| **SPO2** |  | | | | | | |
| **Before ChipEXO™** | 90.0(80.0-95.5) | 91.0(87.5-96.0) | 94.0(91.5-97.5) | 91.0(82.0-93.0) | 95.0(92.5-96.5) | 454.50(430.50-466.25) | **0.016** |
| **After ChipEXO™** | 95.0(86.0-97.0) | 93.0(91.5-96.5) | 95.0(90.5-97.0) | 95.0(87.0-97.0) | 96.0(93.0-98.0) | 468.00(457.25-478.00) | 0.231 |
| ***P*** | 0.248 | 0.417 | 0.894 | 0.068 | 0.475 | 0.249 |  |
| **PCO2** |  | | | | | | |
| **Before ChipEXO™** | 36.0(34.0-42.0) | 37.0(33.5-40.0) | 37.0(32.0-40.5) | 38.0(33.0-42.0) | 36.0(33.0-41.5) | 185.00(172.00-204.75) | 0.524 |
| **After ChipEXO™** | 39.0(34.5-40.5) | 35.0(33.0-39.5) | 37.0(31.0-39.0) | 35.0(32.5-39.5) | 37.0(31.0-41.0) | 186.00(171.00-195.00) | 0.492 |
| ***P*** | 0.553 | 0.361 | 0.754 | 0.270 | 0.530 | 0.221 |  |
| **PO2/FİO2** |  | | | | | | |
| **Before ChipEXO™** | 90.0(62.5-144.0) | 89.0(70.0-124.5) | 103.0(78.5-125.0) | 107.0(81.5-175.0) | 100.0(80.0-164.0) | 505.00(387.25-683.25) | 0.366 |
| **After ChipEXO™** | 112.0(81.0-179.5) | 112.0(72.5-178.0) | 113.0(85.5-193.5) | 180.0(98.0-195.5) | 130.6(120.5-182.5) | 625.50(578.00-804.00) | 0.220 |
| ***P*** | **0.006** | **0.016** | **0.001** | **0.016** | **0.019** | **0.002** |  |
| **PH** |  | | | | | | |
| **Before ChipEXO™** | 7.44(7.43-7.47) | 7.47(7.44-7.49) | 7.45(7.42-7.51) | 7.48(7.45-7.51) | 7.45(7.43-7.47) | 37.25(37.19-37.38) | **0.038** |
| **After ChipEXO™** | 7.45(7.44-7.48) | 7.48(7.43-7.50) | 7.46(7.43-7.49) | 7.49(7.44-7.54) | 7.45(7.39-7.49) | 37.31(37.22-37.36) | 0.439 |
| ***P*** | 0.344 | 0.097 | 0.782 | 0.694 | 0.665 | 0.345 |  |
| **BE** |  | | | | | | |
| **Before ChipEXO™** | 1.80(0.65-2.95) | 1.80(1.05-4.10) | 3.70(1.40-5.15) | 2.40(1.70-6.20) | 2.20(0.50-2.80) | 12.05(10.43-16.28) | 0.197 |
| **After ChipEXO™** | 2.30(1.70-3.80) | 2.30(1.55-3.50) | 3.10(2.15-5.15) | 2.60(1.00-6.15) | 1.60(0.90-3.35) | 12.20(9.60-18.53) | 0.124 |
| ***P*** | 0.152 | 0.638 | 0.727 | 0.861 | 0.624 | 0.552 |  |
| **HCO3** |  | | | | | | |
| **Before ChipEXO™** | 26.20(23.75-26.95) | 26.00(24.65-28.00) | 26.90(24.95-28.60) | 26.20(24.30-28.55) | 26.70(24.45-27.30) | 132.05(126.20-133.80) | 0.724 |
| **After ChipEXO™** | 26.20(24.85-27.20) | 26.00(24.65-27.55) | 27.40(25.90-28.70) | 26.00(24.80-27.75) | 25.80(24.30-27.45) | 130.25(128.55-135.15) | 0.665 |
| ***P*** | 0.529 | 0.861 | 0.916 | 0.239 | 0.463 | 0.753 |  |
| **LACTATE** |  | | | | | | |
| **Before ChipEXO™** | 2.30(1.70-2.75) | 2.10(1.25-2.75) | 2.20(1.30-2.60) | 1.90(1.65-2.20) | 2.20(1.65-2.75) | 11.00(8.90-12.08) | 0.602 |
| **After ChipEXO™** | 2.30(1.50-3.15) | 2.00(1.55-2.30) | 2.10(1.80-2.65) | 1.90(1.35-2.20) | 1.60(1.45-2.90) | 12.15(8.80-13.08) | 0.652 |
| ***P*** | 0.944 | 0.253 | 0.937 | 0.178 | 0.157 | 0.701 |  |
| **Values are expressed median (1^st^-3^rd^ quartiles). Statistically significant *p* values are shown in bold. Different superscripts in the same row indicate a statistically significant difference between groups.** **AUC: Area under the curve** | | | | | | | |
